# Abiotic Stress-Induced *Actin-Depolymerizing Factor 3* From *Deschampsia antarctica* Enhanced Cold Tolerance When Constitutively Expressed in Rice

**DOI:** 10.3389/fpls.2021.734500

**Published:** 2021-09-28

**Authors:** Mi Young Byun, Li Hua Cui, Andosung Lee, Hyung Geun Oh, Yo-Han Yoo, Jungeun Lee, Woo Taek Kim, Hyoungseok Lee

**Affiliations:** ^1^Division of Life Sciences, Korea Polar Research Institute, Incheon, South Korea; ^2^Division of Life Science, Department of Systems Biology, Yonsei University, Seoul, South Korea; ^3^Institute of Life Science and Biotechnology, Yonsei University, Seoul, South Korea; ^4^Polar Science, University of Science and Technology, Daejeon, South Korea

**Keywords:** abiotic stress, actin cytoskeleton, Antarctic, *Deschampsia antarctica* actin-depolymerizing factor 3, low temperature, polar adaptation

## Abstract

The Antarctic flowering plant *Deschampsia antarctica* is highly sensitive to climate change and has shown rapid population increases during regional warming of the Antarctic Peninsula. Several studies have examined the physiological and biochemical changes related to environmental stress tolerance that allow *D. antarctica* to colonize harsh Antarctic environments; however, the molecular mechanisms of its responses to environmental changes remain poorly understood. To elucidate the survival strategies of *D. antarctica* in Antarctic environments, we investigated the functions of actin depolymerizing factor (ADF) in this species. We identified eight *ADF* genes in the transcriptome that were clustered into five subgroups by phylogenetic analysis. DaADF3, which belongs to a monocot-specific clade together with cold-responsive ADF in wheat, showed significant transcriptional induction in response to dehydration and cold, as well as under Antarctic field conditions. Multiple drought and low-temperature responsive elements were identified as possible binding sites of C-repeat-binding factors in the promoter region of *DaADF3*, indicating a close relationship between DaADF3 transcription control and abiotic stress responses. To investigate the functions of DaADF3 related to abiotic stresses *in vivo*, we generated transgenic rice plants overexpressing *DaADF3*. These transgenic plants showed greater tolerance to low-temperature stress than the wild-type in terms of survival rate, leaf chlorophyll content, and electrolyte leakage, accompanied by changes in actin filament organization in the root tips. Together, our results imply that DaADF3 played an important role in the enhancement of cold tolerance in transgenic rice plants and in the adaptation of *D. antarctica* to its extreme environment.

## Introduction

The Antarctic monocot plant *Deschampsia antarctica* Desv. has a considerably wider habitat and larger numbers of populations than *Colobanthus quitensis*, the Antarctic dicot plant (Komárková et al., 1985). *D. antarctica* is highly sensitive to climate change and has shown rapid population increases and enhanced sexual reproduction success rates (Fowbert and Smith, 1994) during regional warming in the Antarctic Peninsula (Smith, 2003). Several studies have focused on the physiological and biochemical changes that allow *D. antarctica* to colonize harsh Antarctic environments. *D. antarctica* exhibits specialized morphological properties, such as distinct leaf anatomy and chloroplast ultra-structure, that influence its photosynthetic efficiency in extreme environments (Giełwanowska et al., 2005; Sáez et al., 2019), and its tolerance to ultraviolet B-induced oxidative stress is enhanced by a combination of phenolic molecules synthesis and the activation of both enzymatic and non-enzymatic antioxidant systems (Köhler et al., 2017). The transcriptome responses of *D. antarctica* have been examined under various abiotic stress conditions (Lee et al., 2013, 2014). Increases in ice recrystallization inhibition protein (*DaIRIP*) expression and recrystallization inhibition activity in response to freezing stress may contribute to the cryotolerance of *D. antarctica* (John et al., 2009). In particular, the transcription factor genes *DaCBF4* and *DaCBF7*, which regulate the expression of cold-regulated genes, can enhance plant cold tolerance when ectopically expressed in rice (Byun et al., 2015, 2018). *DaGolS2*, which encodes a galactinol synthase that is the key enzyme mediating raffinose family oligosaccharide synthesis, improves the resistance of transgenic rice to both cold and drought (Cui et al., 2020). These findings imply that *D. antarctica* could be a model for studying the genetic and metabolic mechanisms of plant adaptations to various abiotic stresses; however, the molecular mechanisms of its adaptation to Antarctic environments remain poorly understood.

The actin cytoskeleton performs diverse cellular processes. The three-dimensional formation, disassembly, and dynamics of actin structure are regulated by diverse actin-binding proteins (Bamburg, 1999; Lappalainen, 2016). One such protein, actin-depolymerizing factor (ADF), is composed of a single folded ADF homology domain. The enzyme activity of ADF influences actin filament turnover mainly by severing actin filaments (Carlier et al., 1997). The functions of ADF/cofilins are modulated by several factors, such as pH (Wioland et al., 2018), phosphorylation (Meng et al., 2004), and interaction with other actin-binding proteins (Xiang et al., 2007). Most vertebrates have one ADF and two cofilins, which are divided into muscle and non-muscle cofilins (Ono et al., 1994). *Xenopus* frogs express two ADF/cofilins, whereas only one ADF/cofilin gene is found in *Caenorhabditis elegans* (Maciver and Hussey, 2002). In contrast, plants have many more ADF/cofilin genes than animals; these are classified into multiple subgroups according to their sequence homology (Huang et al., 2020).

ADFs participate in a vast number of cellular processes, such as cell mobility (Tang and Gerlach, 2017), cytokinesis (Li et al., 2015), and plant cell growth (Hussey et al., 2006). The roles of ADF have been studied extensively *in vivo* among plants with large numbers of ADF genes; notably, the important roles of actin filament structures in pollen development have been analyzed in several studies (Smertenko et al., 2001; Allwood et al., 2002; Xiang et al., 2007; Jiang et al., 2019). In addition, the pollen-specific roles of AtADF7 and AtADF10 during male gametophyte development have been examined based on their spatial and temporal expression, revealing functional redundancy (Bou Daher et al., 2011). Several studies have demonstrated ADF functions in pathological responses (Inada, 2017). Actin polymerization is necessary for increasing actin filament density, which is linked to host plant susceptibility to pathogenic and non-pathogenic bacteria (Henty-Ridilla et al., 2013). The actin cytoskeleton participates in the regulation of stomatal movement, showing different patterns during stomatal opening and closing (Zhao et al., 2011). AtADF5 promotes stomatal closure through the reorganization of actin filaments in response to abscisic acid (ABA) and dehydration stress in a process mediated by the ABA signaling pathway *via* the ABF transcription factor DPBF3, which binds to the ADF5 promoter and turns on its gene expression (Qian et al., 2019). Vegetative profilins and ADFs are specifically upregulated by heat stress, with different expression patterns; actin filament reorganization has been observed in Arabidopsis seedlings treated with heat stress (Fan et al., 2016). Mechanical wounding has been shown to increase microfilament bundles, whereas hypoxia triggers a rapid decrease in polymerized actin and drastic inhibition of protein synthesis in potato, implying that actin cytoskeleton organization may affect translational activity under stress conditions in potato (Morelli et al., 1998).

In maize, 13 *ZmADF* genes showed diverse expression patterns in various tissues in response to different stimuli, including abiotic and phytohormone stresses, implying their specific roles in plant growth, development, and external stimulus responses (Huang et al., 2020). Wheat active ADF protein was induced during cold acclimation, implying a correlation with an increased freezing tolerance in freezing-tolerant wheat cultivars (Ouellet et al., 2001). *OsADF3* expression was upregulated in root tissues in response to ABA or abiotic stress treatment. Transgenic Arabidopsis plants overexpressing *OsADF3* showed enhanced tolerance to dehydration stress, accompanied by the upregulation of several drought tolerance response genes (Huang et al., 2012). The association of these ADF functions with exogenous stimuli, especially abiotic stresses, indicates the crucial roles of these proteins in plants under unfavorable conditions.

In this study, we investigated the functions of an ADF in *D. antarctica*, which has adapted and survived harsh Antarctic environments. Eight *ADF* genes were identified in the transcriptome, and *DaADF3* was selected based on transcriptional responses under environmental stress conditions. To investigate the functions of *DaADF3* related to cold stress, we generated transgenic rice plants overexpressing *DaADF3* and analyzed their phenotypes under low-temperature stress.

## Materials and Methods

### Plant Materials and Growth Conditions

*Deschampsia antarctica* was collected near the King Sejong Antarctic Station (62°14'29"S; 58°44'18"W) on the Barton Peninsula of King George Island in January 2007. The plants were transplanted, cultured *in vitro* in a tissue culture medium consisting of half-strength Murashige and Skoog (MS; Duchefa Biochemie, Haarlem, The Netherlands, 2% sucrose, and 0.8% phytoagar; pH 5.7) under a 16-h/8-h light/dark (long-day) photoperiod with a light intensity of 150μmolm^−2^ s^−1^ at 15°C, and transferred to new medium every 3weeks. Dry seeds from japonica rice (*Oryza sativa* L. cv. dongjin) were washed with 70% ethanol and then sterilized with 0.4% NaClO solution. Sterilized seeds were germinated and grown on half-strength MS medium supplemented with 3% sucrose and 0.75% phytoagar (pH 5.7) for 10–12days. Germinated seedlings were transplanted to soil and grown in a greenhouse at 25–30°C under a long-day photoperiod.

### Identification and Analysis of ADF Family Genes in *D. antarctica*

Rice ADF family members were searched in the Phytozome 13 *Oryza sativa* v7.0 database.^1^ Rice ADF protein sequences were used as queries to search homologous sequences using the BLASTX program and previously reported *D. antarctica* transcriptome assembly data (Lee et al., 2013). Redundant sequences were discarded by manual curation. The Pfam and SMART software packages^2^ were used to confirm the presence of the ADF domain (SM000102). The protein isoelectric point (PI) and molecular weight (Mw) of each gene product were calculated using the ExPasy Compute pI/Mw tool.^3^ Subcellular localization of the identified proteins was analyzed using the ProtComp v9.0 program of the Softberry web tool.^4^ The Multiple EM for Motif Elicitation (MEME^5^) online software was used to identify possible conserved motifs in the amino acid sequences of DaADF proteins. Putative *cis*-regulatory elements around 2000-bp upstream of the start codon of the *DaCBF3* genomic sequence (NCBI accession no. MW818101) were identified using the PlantCARE web tool.^6^ The NCBI BLAST search tool^7^ was used to find DaADF homologous sequences from other plants and to compare the identity among the eight selected DaADF proteins. The NCBI accession numbers of the eight DaADFs are listed in Table 1.

**Table 1:** Detailed information of 8 actin-depolymerizing factor (ADF) family genes identified in this study.

Gene name	Group	NCBI accession	CDS length (bp)	Amino acids (aa)	Isoelectric point	Molecular weight (kDa)	GRAVY	ProtComp
DaADF1	C	MW818093	420	139	5.28	15.94	−0.371	Cytoplasm and Nucleus
DaADF2	B	MW818094	438	145	5.43	16.78	−0.467	Cytoplasm and Nucleus
DaADF3	E	MW818095	429	142	4.41	14.74	−0.263	Cytoplasm and Nucleus
DaADF4	E	MW818096	429	142	5.42	16.14	−0.574	Cytoplasm and Nucleus
DaADF5	A	MW818097	432	143	8.73	16.40	−0.300	Cytoplasm and Nucleus
DaADF7	D	MW818098	420	139	5.91	15.96	−0.381	Cytoplasm and Nucleus
DaADF9	C	MW818099	420	139	5.62	16.08	−0.540	Cytoplasm and Nucleus
DaADF10	B	MW818100	465	154	5.22	17.45	−0.478	Cytoplasm and Nucleus

CDS, coding sequence; bp, base pair; kDa, kilo Dalton; GRAVY, grand average of hydropathy.

### Phylogenetic Analysis

The amino acid sequences of DaADFs and ADF homologs from monocot crops, eudicots, and bryophytes were retrieved from the GenBank database and proofread. Phylogenetic tree was constructed from the datasets using the neighbor-joining method based on the JTT matrix-based model using the MEGA X software (Kumar et al., 2018). All positions with <95% site coverage were eliminated. We allowed <5% alignment gaps, missing data, and ambiguous bases at any position. Support for internal branches was tested using bootstrap analyses with 1,000 replicates. The GenBank accession numbers used are listed in Supplementary Table 1.

### Stress Treatment

For low-temperature treatment, *D. antarctica* plants grown at 15°C were transferred to a climate chamber at 4°C for various time periods (1h to 7days). For dehydration treatment, plants were transferred to filter paper, dried at 15°C, and collected at different time points (1, 2, and 4h) after the imposition of stress. For each treatment, at least two plants were used per biological replicate and a total of three biological replicates per treatment were collected. All sampling for expression analysis was conducted at the same time to avoid variation according to circadian rhythm.

### Real-Time Quantitative Reverse-Tanscription Polymerase Chain Reaction

Total RNA was extracted from mature leaves of *D. antarctica* and rice plants using the RNeasy Plant Mini Kit (Qiagen, Hilden, Germany). The quantity and quality of RNAs were determined using an ND-1000 spectrophotometer (NanoDrop Technologies, Wilmington, DE, United States). First-strand cDNA was synthesized from 2μg total RNA using TOPscript reverse transcriptase (Enzynomics, South Korea) and oligo (dT) primers. RT-qPCR analysis was performed in 20-μl reaction mixtures consisting of 1μl 1:10 diluted cDNA template, 2μM of each primer, and 10μlTB Green Premix Ex Taq (TaKaRa, Japan). The amplification procedure was as follows: denaturation and enzyme activation at 95°C for 5min, followed by 40cycles at 95°C for 10s, 55°C for 10s, and 72°C for 15s. The *DaEF1a* gene was used as an internal control. The DNA sequences of primers used for PCR amplification are listed in Supplementary Table 2.

### Subcellular Localization Assay

The 3' end of the *DaADF3* coding region was tagged with synthetic green fluorescent protein (*sGFP*) in-frame and inserted into the pBI221 binary vector containing the 35S CaMV promoter. The *35S:DaADF3* construct was expressed in the *D. antarctica* protoplast using the PEG*-*mediated DNA transfer method. Fluorescent signals were visualized by fluorescence microscopy (BX51, Olympus, Japan). We used *35S:sGFP* as a cytosolic marker.

### Generation of *DaADF3*-Overexpressing Transgenic Rice Plants

Full-length coding region of *DaADF3* was ligated into pGA2897 binary vector plasmids containing the maize *Ubiquitin* promoter (*Ubi*). The *Ubi:DaADF3* recombinant construct was transformed into *Agrobacterium tumefaciens* strain LBA4404 *via* electroporation as previously described (Park et al., 2016). All rice transformation procedures followed established protocols as recently described by Cui et al. (2018). Generated transgenic rice T0 plants were transplanted in soil under greenhouse conditions and further propagated under paddy field conditions. The harvested transgenic seeds of individual plant were germinated in the half-strength MS medium supplemented with hygromycin B (40mgL^−1^) to select the homozygous T2 generation. Homozygous T3 *DaADF3-*overexpressing (independent lines #A, #B, and #C) transgenic rice progeny were used for our phenotypic analysis.

### Phenotype Analysis of Wild-Type (WT), *Ubi:DaADF3* Rice Plants

For low-temperature stress treatment, 5-week-old rice plants grown at 28°C in the same pot under a long-day photoperiod were transferred to a cold room at 4°C. After 7days of cold treatment, the plants were recovered at 28°C for 20–25days and their growth patterns were monitored as recently described (Cui et al., 2018). Plants that resumed growth with green, healthy leaves were regarded as having survived; the survival rates were determined at 1month of recovery. Data were obtained from at least six biologically independent experiments. Electrolyte leakage analysis was conducted using 8-day-old whole seedlings at different cold treatment time points (0, 5, and 10days) at 4°C. The cold stress-treated seedlings of WT and transgenic rice plants were soaked in a test tube containing 35ml distilled water on an orbital shaker (200rpm) at room temperature overnight. The total leaf chlorophyll (chlorophyll a+chlorophyll b) content of WT and transgenic rice plants was measured before and after stress treatment as described by Min et al. (2016) with slight modifications. The amounts of chlorophyll a+chlorophyll b were measured at 664.2nm and 648.6nm, respectively, using an enzyme-linked immunosorbent assay microplate reader (VERSAmax, Molecular Devices, United States) and normalized to the dry weight of the leaves of each genotype. The electrolyte conductivity of each sample was determined following the method of Min et al. (2016). Light-grown 8-day-old whole seedlings of WT and *DaADF3* overexpressors were incubated at 4°C for 0, 5, and 10days. Then, the seedlings were soaked in 35ml distilled water at 25°C overnight, and electrolyte leakage was measured before and after autoclaving using a conductivity meter (Orion Star A212, Thermo Scientific, United States).

### Visualization of Actin Dynamics Through Fluorescence Microscopy

Immunofluorescence experiments were conducted to detect G-actin monomers and F-actin filaments as previously described (He et al., 2006). For G-actin and F-actin labeling, root tissues were fixed by vacuum infiltration for 5min in a freshly prepared solution of 1% formaldehyde in phosphate-buffered saline (PBS), kept in the fixation solution for 1h, and then washed in PBS three times. Then, 1-cm pieces of the root apices were cut and incubated in a mixture of Alexa Fluor 488-DNase I (Invitrogen) and Alexa Fluor 568-phalloidin (Invitrogen) in PBS (0.137M NaCl, 2.7mM KCl, 1.8mM KH_2_PO_4_, 10mM Na_2_HPO_4_, pH 7.4). After 10min, the samples were washed five times with PBS and observed using a cooled charge-coupled device camera with a laser scanning confocal microscope (LSM510 META; Carl Zeiss, Germany). The G-actin and F-actin signals were excited by lasers at 488 and 550nm, respectively. The fluorescence signals were measured using a sliding 0.3-μm detection window in a confocal microscope.

## Results

### Identification of *ADF* Genes in *D. antarctica* Transcriptome

To identify the ADF gene family members in *D. antarctica*, we searched a previously reported transcriptome database (Lee et al., 2013) using the BLASTX program with rice ADF proteins as queries and used the SMART web tool to determine whether the ADF domain (SM000102) was present in the candidate ADF genes. After manual curation, eight ADF domain-containing genes were identified; these eight genes were designated as *D. antarctica ADF* (*DaADF*) and the corresponding proteins were named DaADF. A serial number was assigned to each gene according to the corresponding rice homolog with the highest similarity. The predicted size of DaADFs ranged from 139 to 154 amino acids, the isoelectric point ranged from 4.41 to 8.73, and the molecular weight ranged from 14.74 to 17.45kDa, and the grand average of hydropathy (GRAVY) values of all DaADF were<0, indicating that they are hydrophilic. Subcellular localization of the proteins was predicted to be multi-localized proteins, in the cytoplasm and nucleus (Table 1). The DaADFs shared >80% sequence identity with *Triticum aestivum* and other monocot ADFs, ranging from 75 to 98% (Supplementary Table 3). The sequence identity among the eight DaADFs ranged from 39 to 72% (Supplementary Table 4).

### Phylogenetic Analysis of the DaADF Family

**Figure 1:**
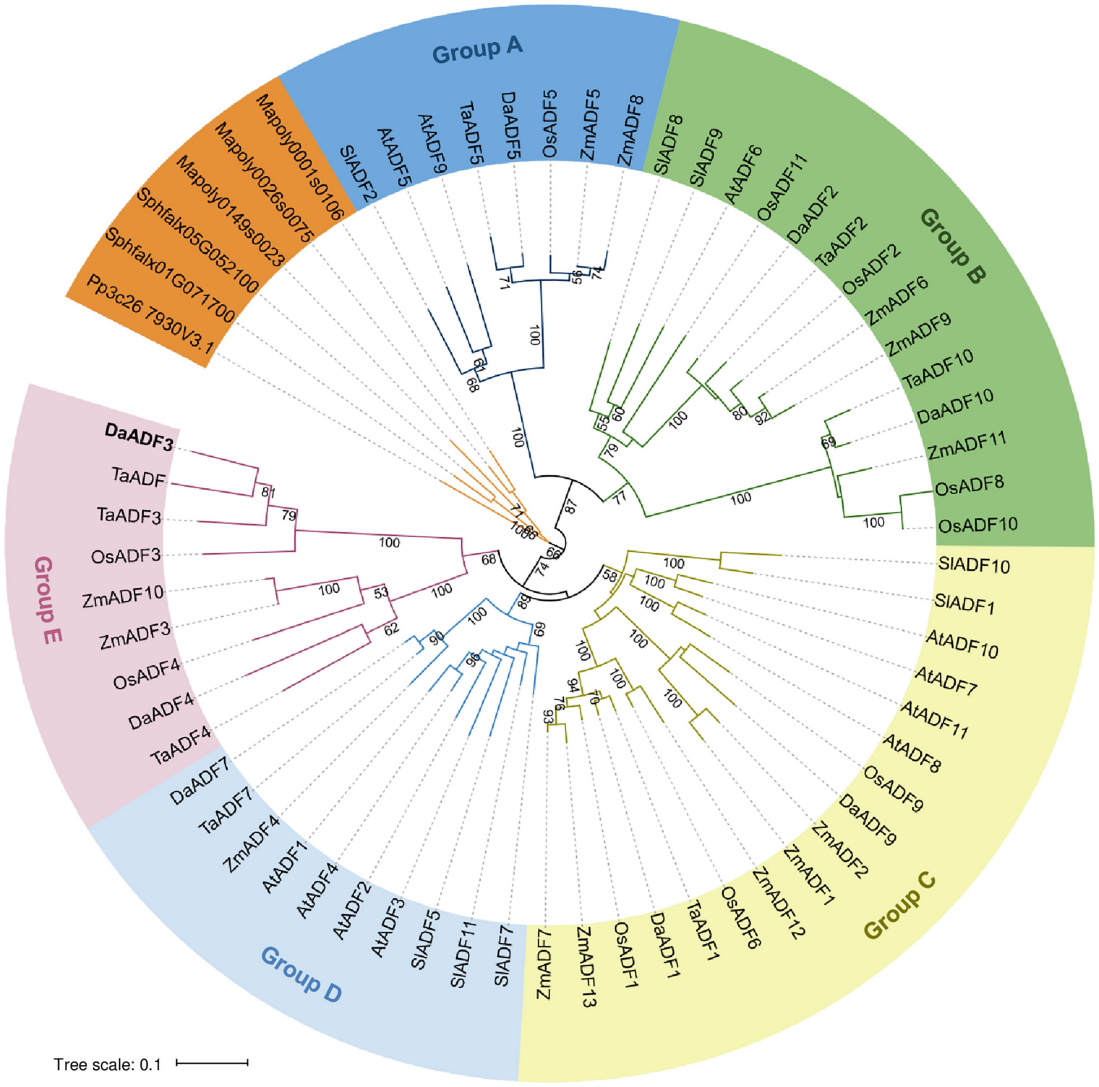
Phylogenetic analysis of actin depolymerizing factor (ADF) proteins. The full-length amino acid sequences of DaADFs and homologs from other plants and algae were retrieved from the GenBank database and proofread. Phylogenetic trees were constructed from the datasets using the neighbor-joining method based on the JTT matrix-based model using the MEGA X software. Evolutionary distances were computed using the JTT matrix-based method. The ADF members were divided into five groups (A to E).

To investigate the phylogenetic relationships among the DaADF family, we selected five flowering plants as references, including *Arabidopsis thaliana*, *Solanum lycopersicum*, *Oryza sativa*, *Triticum aestivum*, and *Zea mays*, as well as three bryophytes as an outgroup: *Marchantia polymorpha*, *Physcomitrium patens*, and *Sphagnum fallax* (Supplementary Table 1). The DaADFs were distributed in five subfamilies (Groups A to E), excluding the outgroup. DaADF5 clustered in Group A, DaADF2 and DaADF10 clustered in Group B, DaADF1 and DaADF9 clustered in Group C, DaADF7 clustered in Group D, and DaADF3 and DaADF4 clustered in Group E (Figure 1, and Table 1). The conserved motifs of DaADF family proteins were further identified using the MEME online software (Supplementary Figure 1), which predicted that DaADF family proteins contain at least four conserved motifs. Six of the eight DaADF proteins have an arrangement of motifs (order, 3–2–1-4), whereas motif 4 was missing from DaADF3 and DaADF4 in Group E, which had motif arrangements of 3–2–1-5 and 3–2–1-7, respectively. In summary, the overall structure of the DaADF family is highly conserved and relatively stable; however, we assume that DaADF3 and DaADF4, which belong to Group E, underwent an independent differentiation process during evolution.

### Expression Analysis of *DaADF* Genes in the Antarctic Field

**Figure 2:**
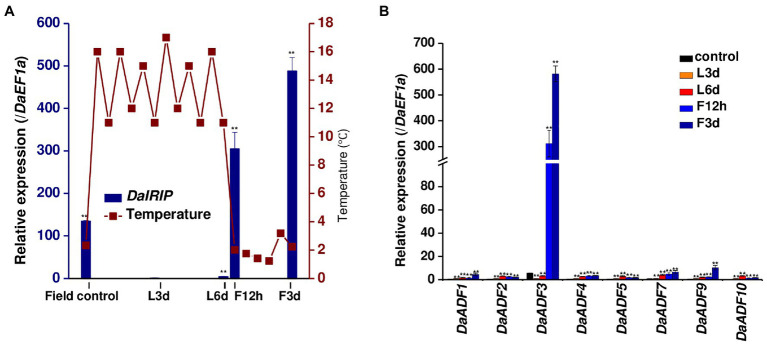
Expression profiles of *DaADF* homologs in response to different growth conditions in the Antarctic field and the laboratory. **(A)** Expressional changes of *DaIRIP* in response to different growth conditions. *Deschampsia antarctica* plants were collected in Antarctica (Field control) and incubated under laboratory conditions for 3days (L3d) and 6days (L6d), and then transferred back to the Antarctic field for 12h (F12h) and 3days (F3d). **(B)** Expression patterns of eight *DaADF* genes in response to the Antarctica field and laboratory conditions in *D. antarctica* plants. Total RNA was isolated from each sample and used for reverse-transcription quantitative polymerase chain reaction (RT-qPCR) analysis using a gene-specific primer set (Supplementary Table 2). The relative expression of each gene was normalized to that of *DaEF1a* as an internal control. Data are means ± standard deviation (SD) of three biologically independent experiments (^**^p<0.01, Student’s *t*-test).

We performed gene expression analysis of the eight selected *DaADF* genes in *D. antarctica* samples previously collected in Antarctica (Cui et al., 2020) to identify genes related to adaptation to the Antarctic environment. Briefly, naturally colonized *D. antarctica* plants (field control) were collected on the Barton Peninsula of King George Island and incubated in the laboratory (15°C, long-day photoperiod) for 6days (L3d and L6d; Figure 2A). The plants were transferred back to an Antarctic field and harvested at different time points (12h and 3days). When the plants were transported from the field to the laboratory, the expression of *DaIRIP*, a cold-induced gene in *D. antarctica* (John et al., 2009), declined to the background level. However, the transcript levels of *DaIRIP* rapidly increased after the plants were transferred back to the field (from 12h to 3days; Figure 2A). Expressional changes in the eight *DaADF* genes under the same experimental conditions showed that the transcripts of *DaADF3* were reduced in samples incubated in the laboratory, and considerably elevated in samples transferred back to the Antarctic field *DaADF3* having a pattern of expressional alterations most similar to that of *DaIRIP* (Figure 2B).

### *DaADF* Gene Expression in Response to Cold and Drought

**Figure 3:**
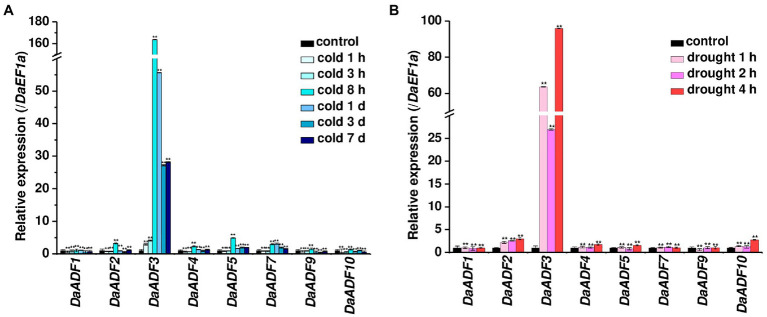
Expression profiles of *DaADF* homologs in response to cold and drought. Laboratory-cultured 3-week-old *D. antarctica* seedlings were subjected to **(A)** cold (4°C for 7days) and **(B)** drought (air-dried on filter paper at 15°C for 4h) treatments, and total RNA prepared from the treated tissues was analyzed by RT-qPCR using gene-specific primer sets (Supplementary Table 2). The relative expression level of each gene was normalized to that of *DaEF1a*. Data are means ± SD of three biologically independent experiments (^**^p<0.01, Student’s *t*-test).

Next, we investigated the expression changes of *DaADF* genes under low-temperature and dehydration treatment, as representative environmental stresses in Antarctica. Each stimulus was applied to *D. antarctica* plants grown at 15°C in the laboratory, and gene expression changes were evaluated by RT-qPCR. The genes *DaADF2*, *DaADF3*, *DaADF4*, *DaADF5*, and *DaADF7* showed higher gene expression under cold stress treatment. Among these, *DaADF3* showed the most significant expressional changes, reaching a peak at 8h and slowly decreasing thereafter (Figure 3A). *DaADF3* also displayed prominent transcriptional changes in response to drought (Figure 3B), showing a nearly 100-fold difference compared to the control condition. In conclusion, among the eight *DaADF* genes identified, *DaADF3* showed the largest changes in gene expression in response to cold and drought stresses. The results shown in Figures 2, 3 imply that *DaADF3* encodes an ADF that plays key roles in the adaptation mechanism of *D. antarctica* for survival in harsh Antarctic environmental conditions.

### Analysis of *Cis*-Acting Elements of DaADF3

In combination with the expression characteristics of *DaADF3*, we analyzed *cis*-acting elements to explore its specificity in response to abiotic stress treatments and to provide evidence for its regulatory control at the transcriptional level. The most prominent *cis*-acting elements were stress-related, including multiple DRE core and ABRE elements (Supplementary Figure 2). This finding indirectly explains the high expression of *DaADF3* under cold and drought stress treatment. The *DaADF3* promoter region also contains light responsive elements, such as AE-box, GATA-motif, and G-box, and several hormone responsive elements, such as AuxRR-core, CGTCA-motif, and TGACG-motif, which are associated with responses to a variety of abiotic stresses.

### Subcellular Localization of DaADF3

**Figure 4:**
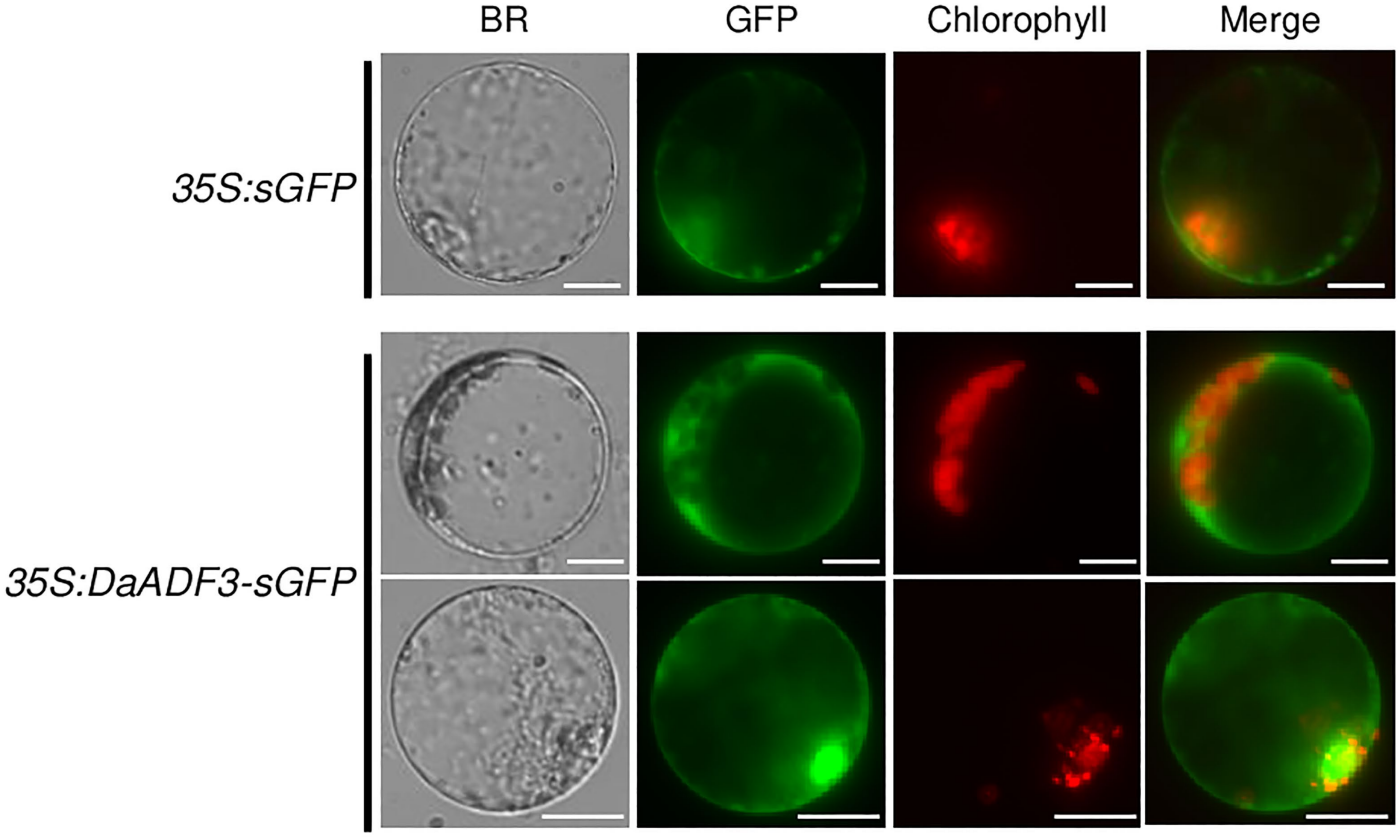
Subcellular localization of DaADF3. The *35S:DaADF3-sGFP* fusion construct was transfected into protoplasts prepared from mature leaves of *D. antarctica*. Fluorescent signals of the expressed proteins were visualized by fluorescence microscopy. sGFP was used as a cytosolic marker protein. Bars=20μm.

Subcellular localization of DaADF3 was investigated *via* a protoplast transient expression system. Protoplasts were prepared from mature leaves of *D. antarctica* plants and transfected with the *35S:DaADF3-sGFP* fusion construct. The expressed proteins were visualized by fluorescence microscopy. Fluorescence signals for DaADF3 were predominantly located in the cytosolic fraction and nucleus (Figure 4), as predicted by the results shown in Table 1.

### Generation of *DaADF3*-Overexpressing Transgenic Rice Plants

**Figure 5:**
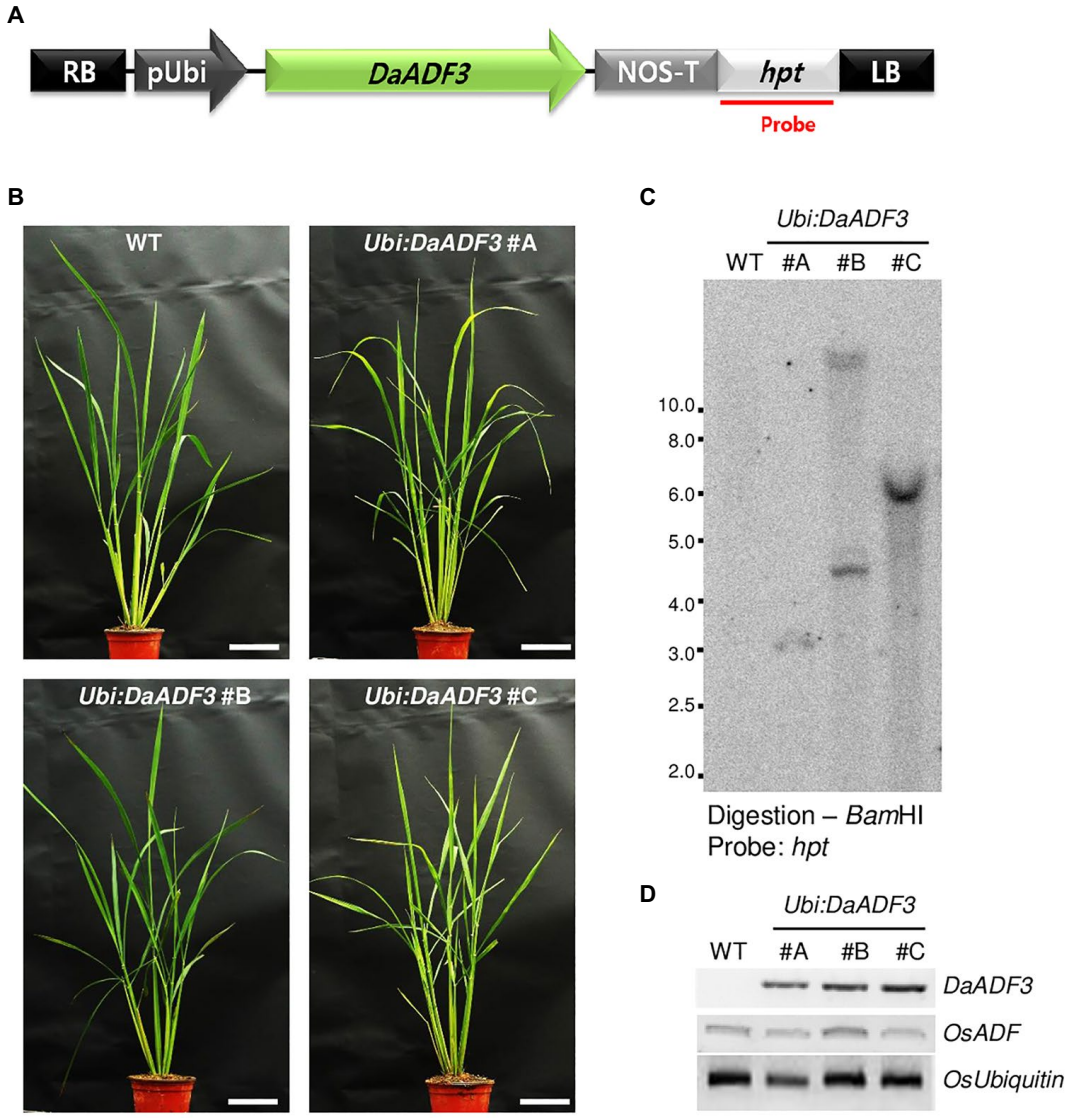
Characterization of *DaADF3*-overexpressing transgenic rice plants. **(A)** Schematic representation of a *DaADF3*-overexpressing binary vector construct. *Hpt*, hygromycin phosphotransferase; LB, left border; pUbi, maize ubiquitin promoter; RB, right border; T-NOS, NOS terminator. **(B)** Morphology of 2-month-old wild-type (WT) and T3 *Ubi:DaADF3* (independent lines #A, #B, and #C) transgenic rice plants grown under a long-day (16-h/8-h light/dark) photoperiod. Bars=10cm. **(C)** Genomic Southern blot analysis. Total leaf genomic DNA was extracted from WT and T3 *Ubi:DaADF3* (lines #A, #B, and #C) rice plants. DNA was digested with *Bam*HI and hybridized to a ^32^P-labeled hygromycin B phosphotransferase (*hpt*) probe. **(D)** RT-PCR analysis of the WT and T4 *Ubi:DaADF3* (lines #A, #B, and #C) rice plants to examine *DaADF3* overexpression. *OsUbiquitin* was used as a loading control.

Although genetic transformation has been widely used to explore the cellular roles of stress-related genes in diverse plant species, stable gene transformation and regeneration of *D. antarctica* have not yet been established. *Deschampsia antarctica* and rice, a monocot model plant, belong to the same Poaceae family. Therefore, to investigate the role of DaADF3 *in planta*, we generated transgenic rice plants that constitutively expressed *DaADF3* under the control of the maize *Ubiquitin* promoter (*Ubi*). Under normal growth conditions, the *DaADF3-*overexpressing T3 transgenic rice plants (*Ubi:DaADF3*) exhibited no detectable morphological differences compared with WT rice plants. Based on the results of genomic Southern blot analysis, three independent *Ubi:DaAD3F* lines (#A, #B, and #C) were selected, in which *DaADF3* transcripts were detected by RT-PCR (Figure 5).

### *Ubi:DaADF3* Plants Exhibited Greater Cold Stress Tolerance Than WT Plants

**Figure 6:**
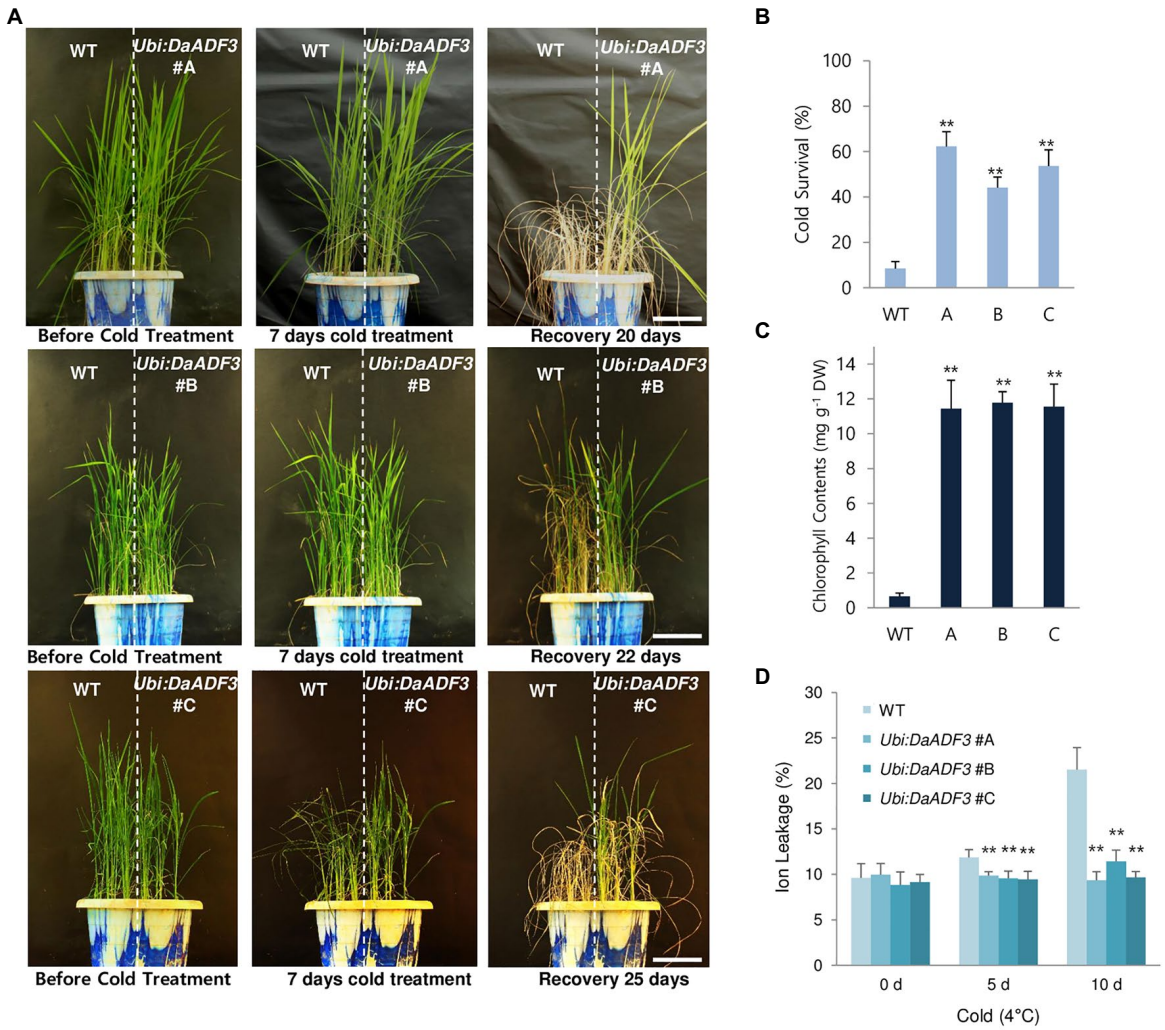
Increased tolerance of *DaADF3*-overexpressing transgenic rice plants in response to cold stress. **(A)** Cold stress phenotypes of WT and *Ubi:DaADF3* transgenic rice plants. Light-grown, 5-week-old WT and T3 *Ubi:DaADF3* (lines #A, #B, and #C) rice plants were transferred to a cold room at 4°C for 8days, after which the plants recovered at 28°C for 20–25days. Bars=15cm. **(B)** Survival rates of WT and *Ubi:DaADF3* plants in response to cold stress. Data are means ± SD (*n*≥6 biologically independent experiments; > 30 plants per assay, ^**^p<0.01, Student’s *t*-test). **(C)** Total leaf chlorophyll content of WT and *Ubi:DaADF3* plants after cold treatment. The amounts of leaf chlorophyll (chlorophyll a+chlorophyll b) were determined 1month after recovery from cold stress. Data are means ± SD (n≥3 biologically independent experiments; > 10 plants per assay, **p<0.01, Student’s *t*-test). **(D)** Electrolyte leakage analysis of WT and *Ubi:DaADF3* plants before and after cold stress. Electrolyte leakage analysis was conducted using 8-day-old WT and *Ubi:DaADF3* (independent lines #A, #B and #C) seedlings that were incubated at 4°C for 0, 5, and 10days. Data are means ± SD (*n*=3 biologically independent experiments; > 12 plants per genotype per experiment, ^**^p<0.01, Student’s *t*-test).

To examine the cold tolerant phenotypes of the *DaADF3* overexpressors, WT and T3 *Ubi:DaADF3* (independent lines #A, #B, and #C) rice plants were grown at 28°C for 5weeks under a long-day photoperiod, transferred to a cold room at 4°C, and incubated under continuous light. After 7days of low-temperature treatment, these plants were transferred back to the growth room at 28°C, allowed to recover and grow for 20–25days, while their survival was monitored. Under our experimental conditions, most of the WT rice plants exhibited discolored leaves with markedly reduced turgor after recovery from cold stress and were unable to grow (survival: 8.4%±3.0%). In contrast, the *DaADF3* overexpressors clearly showed healthier morphology and resumed growth after being relieved from cold stress (survival: 44.1%±4.7 to 62.3%±6.4%; Figures 6A,B).

To measure the total leaf chlorophyll content (chlorophyll a+chlorophyll b), mature leaves were detached from plants of each genotype before and after cold treatment. Before cold treatment, the chlorophyll content of WT and *Ubi:DaADF3* plants was indistinguishable. However, the *DaADF3* overexpressors contained higher amounts of chlorophyll than WT rice plants in response to cold temperature. After 1month of recovery from cold treatment at 4°C, the chlorophyll content of WT leaves was 0.65±0.19mgg^−1^ dry weight (DW), whereas the chlorophyll contents of the *Ubi:DaADF3* plants ranged from 11.4±1.6 to 11.8±0.6mgg^−1^ DW (Figure 6C). To quantify the cellular response to cold stress, an electrolyte leakage assay was performed. WT seedlings showed higher ion leakage rates (11.9±0.8% at 5days and 21.5±2.4% at 10days) than *Ubi:DaADF3* seedlings (9.4±0.9% to 9.9±0.4% at 5days and 9.3±1.0% to 11.4±1.2% at 10days) in response to prolonged cold stress (Figure 6D).

**Figure 7:**
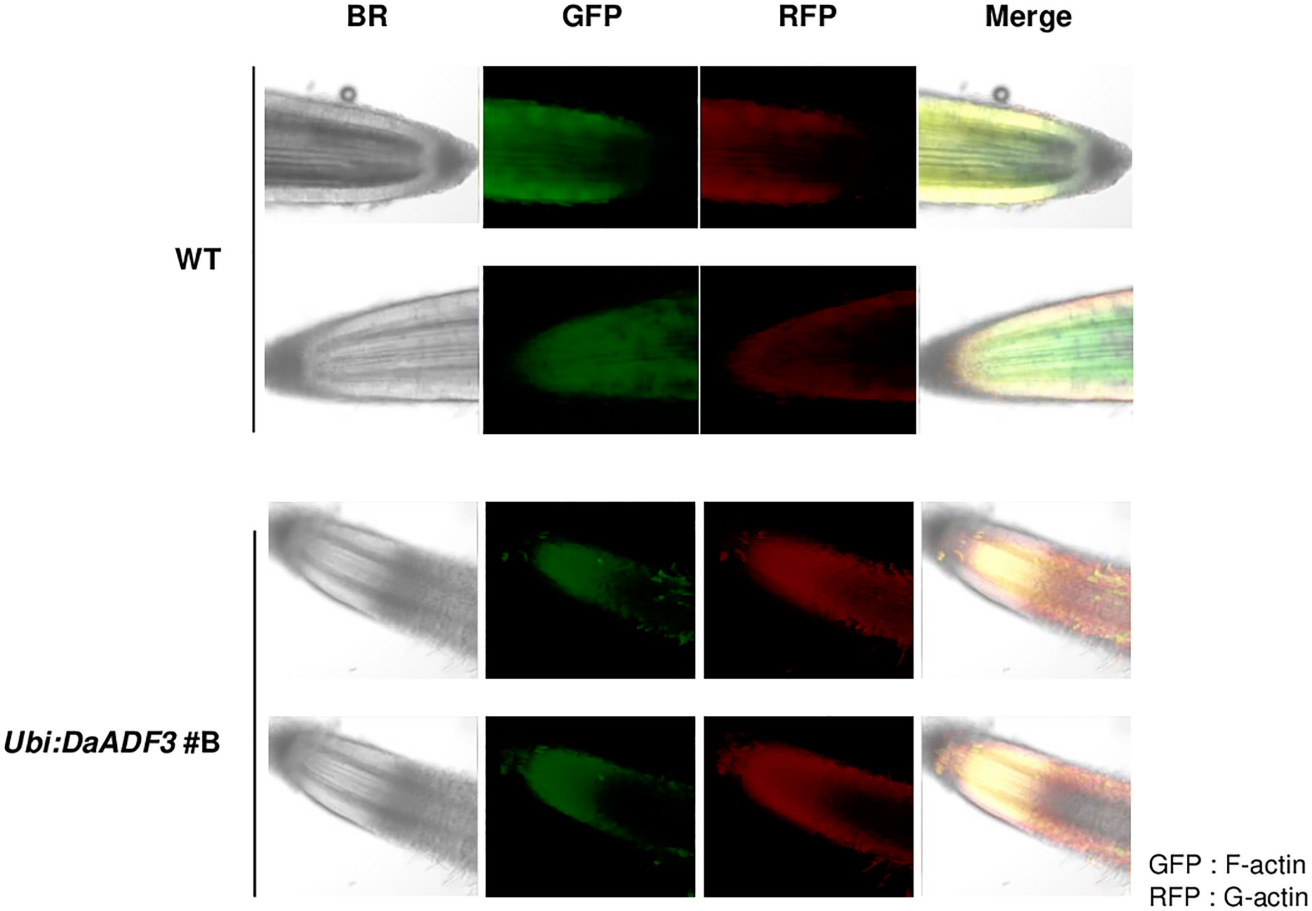
Confocal image of G-actin and F-actin in root tips of WT and *Ubi:DaADF3* transgenic rice plants. Pieces of root tissue apices (1cm) were cut, fixed directly, and incubated in a mixture of Alexa Fluor 488-DNase I and Alexa Fluor 568-phalloidin. The stained samples were observed using a laser scanning confocal microscope. The G-actin and F-actin signals were excited by lasers at 488 (RFP) and 550nm (GFP), respectively.

To further assess the role of DaADF3 in actin cytoskeleton structural dynamics, we observed G-actin and F-actin in the root tips of WT and *Ubi:DaADF3* line #B transgenic rice plants using Alexa Fluor 488-DNase I (G-actin) and Alexa Fluor 568-phalloidin (F-actin) double labeling and laser scanning confocal microscopy. The ratio of G-actin to F-actin was found to be higher in *Ubi:DaADF3* transgenic rice plants than in the WT, which indicate that DaADF3 functions *in planta* to depolymerize F-actin into G-actin in the root tips of transgenic plants (Figure 7).

## Discussion

The *Wcor719* gene, which encodes ADF, has been shown to be rapidly and strongly upregulated by low temperature, with greater accumulation in tolerant winter wheat and rye cultivars than in less tolerant cultivars (Danyluk et al., 1996). Ouellet et al. (2001) detected actin depolymerization activity in the gene product and renamed *Wcor719* as *TaADF*. Based on the induction of an active ADF during cold acclimation and its correlation with increased freezing tolerance, the protein has been suggested to be required for cytoskeletal rearrangements occurring upon exposure to low temperatures. To determine the association between ADF and the strong cold tolerance shown by *D. antarctica*, we performed transcriptome-based screening of the ADF gene family. Unlike most animals, which have only one or two ADF/cofilins genes, plants contain a more expanded ADF gene family; 11 ADF genes have been verified in both *A. thaliana* (Nan et al., 2017) and rice (Huang et al., 2012), and 25 genes were found in the hexaploid wheat genome (Xu et al., 2021). We isolated eight *ADF* genes from *D. antarctica*, fewer than those found in other model plants, perhaps because we used only the transcriptome as a resource since there is presently no reference genome for this species.

ADF proteins from flowering plants have been classified into four main groups to date: rice, Arabidopsis, tomato, and wheat (Feng et al., 2006; Khatun et al., 2016; Huang et al., 2020; Xu et al., 2021). In this study, we phylogenetically classified the *D. antarctica* ADF family with model plants, such as *A. thaliana*, tomato, rice, wheat, and maize, forming five subgroups (A to E; Figure 1). In particular, group E was a monocot-specific clade containing TaADF and DaADF3 as the closest homologs. Similar to *TaADF*, *DaADF3* transcription was rapidly induced by low temperature and environmental stress in the Antarctic field (Figures 2, 3), implying that DaADF3 functions in the adaptation and colonization of *D. antarctica* in response to low temperature. This finding is consistent with a previous report that orthologous ADF gene pairs from the same subgroup have close evolutionary relationships and may preserve biological functions and transcriptional control mechanisms (Huang et al., 2020).

The direct regulation of low temperature-inducible *AtADF5* by CBFs, representative cold stress signaling transcription factors, was recently revealed in Arabidopsis. CBF proteins bind to the promoter and turn on the transcription of *AtADF5*, which is involved in regulating cytoskeleton dynamics, allowing plants to adapt to unfavorable environments (Zhang et al., 2021). *DaADF3* expression increased from the beginning of cold stress treatment, peaked at 8h, and decreased thereafter (Figure 3A). Previous studies have reported that *DaCBF4* and *DaCBF7* transcription was immediately upregulated and soon reduced to normal states following stress treatment (Byun et al., 2015, 2018). The *DaADF3* promoter contains multiple DRE and LTRE elements that are possible binding sites for CBF transcription factors (Supplementary Figure 2), and synthetic oligonucleotides designed based on the DRE element of the *DaADF3* promoter have shown high binding affinity to DaCBF7 (Byun et al., 2015). Thus, we assume that *DaADF3* transcription can be modulated by CBF transcription factors in the Antarctic flowering monocot plant examined in this study, and that DaADF3 further regulates actin cytoskeleton dynamics to participate in the regulation of plant adaptation to the cold Antarctic environment. However, *in vivo* transcriptional regulation of *DaADF3* by CBF transcription factors has not yet been demonstrated. Further experiments, for example, transactivation assay using DaCBF7 and *DaADF3* promoter may provide more evidence for molecular regulation of *DaADF3* associated to cold adaptation of *D. antarctica*.

DaADF3 predominantly localized to the cytosolic fraction and nucleus in protoplasts extracted from *D. antarctica* leaves (Figure 4). This is consistent with previous studies on subcellular localization of ADFs from wheat and cucumber (Liu et al., 2016; Tang et al., 2016; Xu et al., 2021), using the ADF protein with C-terminal GFP driven by the 35S promoter. However, whether these GFP fusion proteins are fully functional in plant cells remains to be determined, because fusion constructs containing cofilin with GFP fused either at its N or C terminus could not complement its loss of function phenotypes in budding yeast (Okreglak and Drubin, 2007) and only intramolecular ADF-GFP fusion constructs were functional in Arabidopsis pollen tube (Zheng et al., 2013).

Ectopic expression of *OsADF3* in Arabidopsis enhanced its tolerance to dehydration stress, accompanied by the upregulation of several drought tolerance response genes (Huang et al., 2012). In contrast, *TaADF16* overexpression increased the freezing tolerance of transgenic Arabidopsis, possibly due to enhanced reactive oxygen species scavenging in cells. The expression levels of seven cold-response genes were found to be upregulated in transgenic Arabidopsis plants (Xu et al., 2021). In this study, transgenic rice overexpressing *DaADF3* showed increased tolerance to low temperature stress compared to WT (Figure 6) and the ectopic expression of *DaADF3* altered the actin cytoskeleton structure of rice plants (Figure 7). The abundance of G-actin relative to F-actin was exhibited to be higher in transgenic rice plants than in the WT, implying enhanced actin depolymerization activity in DaADF3 overexpressing plants. Furthermore, *D. antarctica* seedlings showed cytoskeleton structural changes in response to cold stress treatment (Supplementary Figure 3), which corresponds to the modulation of *DaADF3* transcription under cold stress (Figure 3A). Together, these results imply that DaADF3 regulates the cytoskeleton structure to adapt to changing environmental conditions, especially cold stress in *D. antarctica*.

In this study, we visualized actin filament to investigate the changes of cytoskeleton in response to low temperature stimulus. As shown in Figure 7 and Supplementary Figure 3, the ratio of G-actin to F-actin was modulated in *DaADF3* overexpressing rice plants and cold stress treated *D. antarctica* seedling. This might be linked to the protein activity of DaADF3, the depolymerization of actin filaments in plants. Previous studies have used chemical inhibitors of actin conformational changes, such as actin-disrupting drug latrunculin B and actin-stabilizing drug phalloidin, to elucidate their effects on actin dynamics in Arabidopsis plants in response to *Pseudomonas syringae* pv. *tomato* DC3000 (Henty-Ridilla et al., 2013) and heat stress (Fan et al., 2016). However, the effect of these inhibitors on the cold stress response of rice or *D. antarctica* is still unknown. Further experiments using actin-specific inhibitor may contribute to a comprehensive understanding of the action mechanism of actin cytoskeleton dynamics of *D. antactica* in response to the environmental stresses.

Cytoskeleton reorganization has been shown to be a major component of the signaling pathway under cold stress in alfalfa. The degrees of cell membrane fluidity affected the expression of the cold acclimation gene *cas30*, as well as calcium influx, altering its resistance to freezing stress. During cold acclimation, cytoskeleton reorganization plays a key role in cold stress responses by linking cell membrane rigidification and calcium influx to enhance survival under unfavorable conditions in *Medicago sativa* (Orvar et al., 2000). In a wheat thermosensitive genic male sterile (TGMS) line, male sterility was found to be strictly controlled by temperature. Cold stress repressed the transcription of cytoskeleton dynamic factors, resulting in defective cytokinesis during meiosis I. These findings may explain the induction of male sterility by low temperature in wheat TGMS line, and demonstrate the importance of cytoskeleton dynamics in the reproductive process (Tang et al., 2011). *Deschampsia antarctica* flourishes in harsh Antarctic environment by reproducing through autogamy, with excess pollen production (Yudakova et al., 2016). Cytoskeleton dynamics *via* DaADF3 may be another mechanism of action for *D. antarctica* reproduction, as it is one of only two flowering plants that have adapted to this environment.

Due to the absence of a reference genome and limited applicable genetics tools, studies on the molecular adaptation mechanisms of polar plants are far behind those of model plants. To overcome these practical limitations, we applied a combined approach, including transcriptome analysis and plant genetic transformation, and revealed the function of DaADF3 in plant cold tolerance using transgenic rice overexpressing *DaADF3*. Further molecular and physiological studies will be needed to determine whether CBF is the actual upstream activator that turns on *DaADF3* transcription, and to identify the mechanism by which actin cytoskeleton changes increase plant cold tolerance.

## Data Availability Statement

The datasets presented in this study can be found in online repositories. The names of the repository/repositories and accession numbers can be found in the article/Supplementary Material.

## Author Contributions

WTK and HL conceived and designed the study. MYB, LHC, AL, HGO, and Y-HY performed the experiments. MYB, LHC, and JL analyzed the data. MYB, LHC, WTK, and HL discussed the results and wrote the manuscript. All authors contributed to the article and approved the submitted version.

## Funding

This work was supported by “Post-Polar Genomics Project: Functional genomic study for securing of polar useful genes (PE21160),” funded by Korea Polar Research Institute (KOPRI), “Development of potential antibiotic compounds using polar organism resources (15250103, KOPRI Grant PM21030),” funded by the Ministry of Oceans and Fisheries, Korea, and the Basic Science Research Program, Project No. 2018R1A6A1A03025607 through the National Research Foundation (NRF), funded by the Ministry of Education, Korea.

## Conflict of Interest

The authors declare that the research was conducted in the absence of any commercial or financial relationships that could be construed as a potential conflict of interest.

## Publisher’s Note

All claims expressed in this article are solely those of the authors and do not necessarily represent those of their affiliated organizations, or those of the publisher, the editors and the reviewers. Any product that may be evaluated in this article, or claim that may be made by its manufacturer, is not guaranteed or endorsed by the publisher.

## Footnotes

^1^ https://phytozome-next.jgi.doe.gov/

^2^ http://smart.embl-heidelberg.de/

^3^ https://web.expasy.org/compute_pi/

^4^ http://www.softberry.com/berry.phtml?topic=protcomppl&group=programs&subgroup=proloc

^5^ https://meme-suite.org/tools/meme

^6^ http://bioinformatics.psb.ugent.be/webtools/plantcare/html/

^7^ http://www.ncbi.nlm.nih.gov/BLAST/
