# Supplementary material for: Abiotic Stress-Induced Actin-Depolymerizing Factor 3 From Deschampsia antarctica Enhanced Cold Tolerance When Constitutively Expressed in Rice
Source: Front Plant Sci. 2021 Sep 28;12:734500. doi: 10.3389/fpls.2021.734500 (PMC8506025; doi:10.3389/fpls.2021.734500)
Supplement: Supplementary file 1 [file Data_Sheet_1.docx]

Supplementary Materials


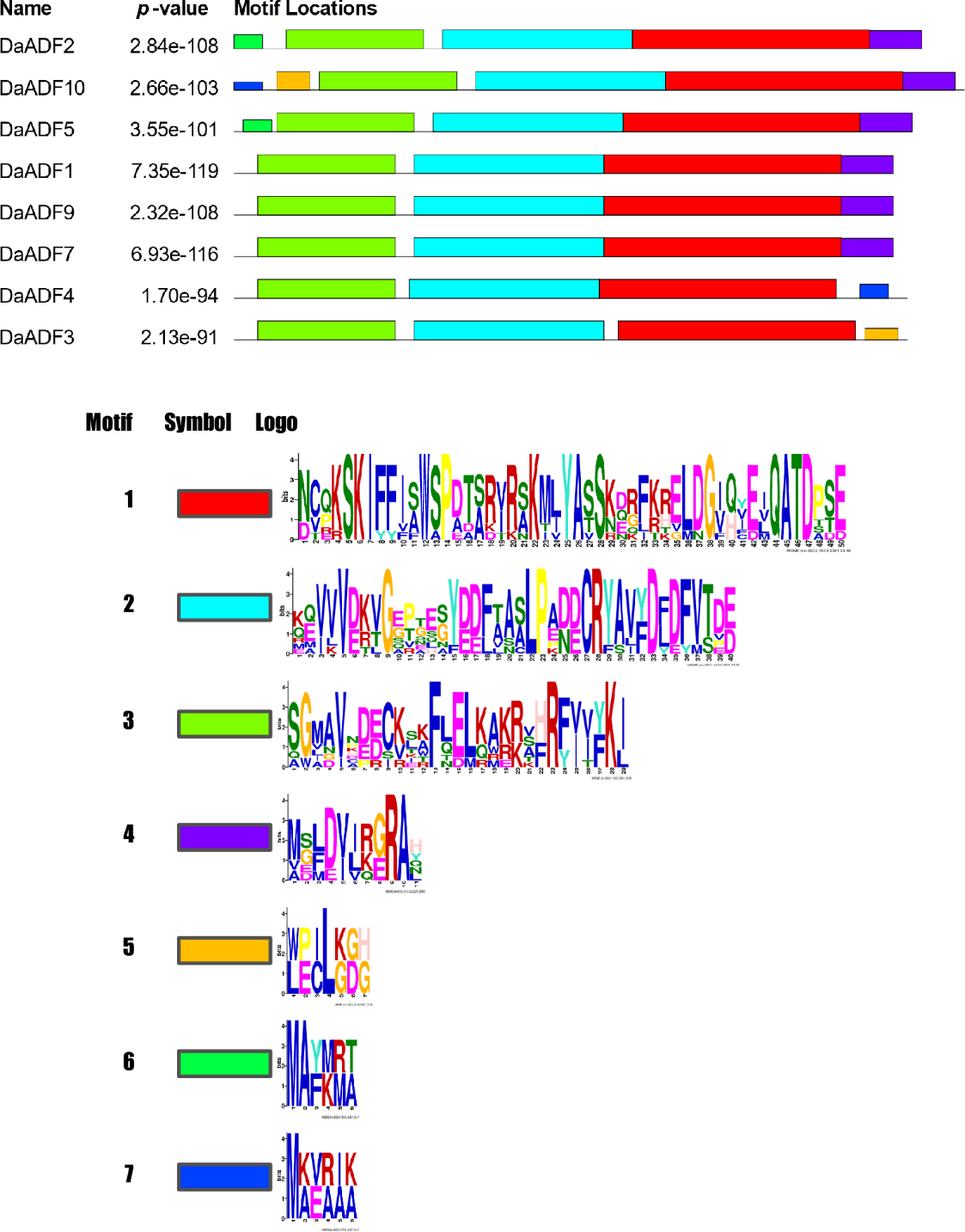


**Supplementary Figure 1.** Schematic representation of the conserved motif identified in *D. antarctica* ADF proteins. Different colors represent different motifs. The motif order in the figure corresponds to their position in the individual protein sequences. The name of each protein member is shown on the left side of the figure.


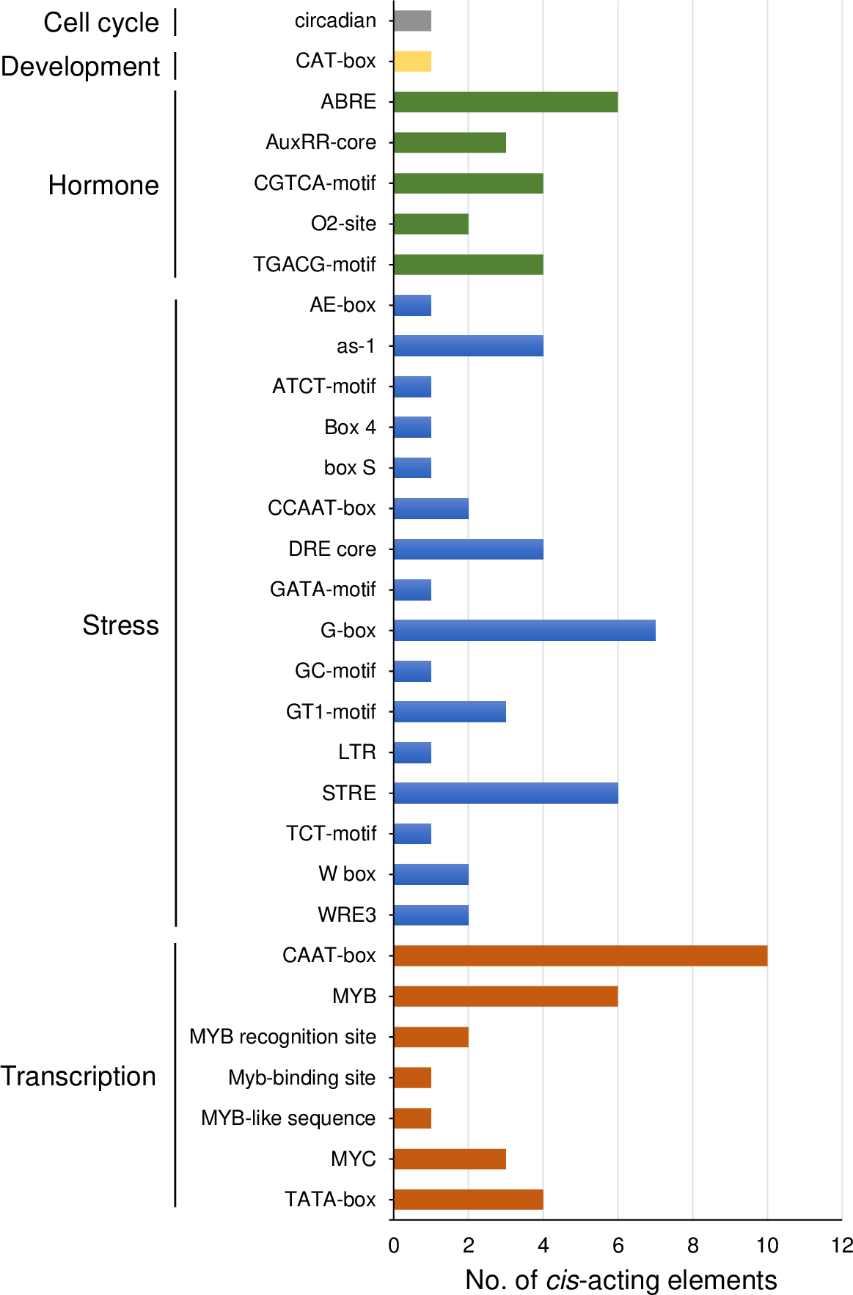


**Supplementary Figure 2.** Analysis of *cis*-acting elements in the *DaADF3* promoter region.


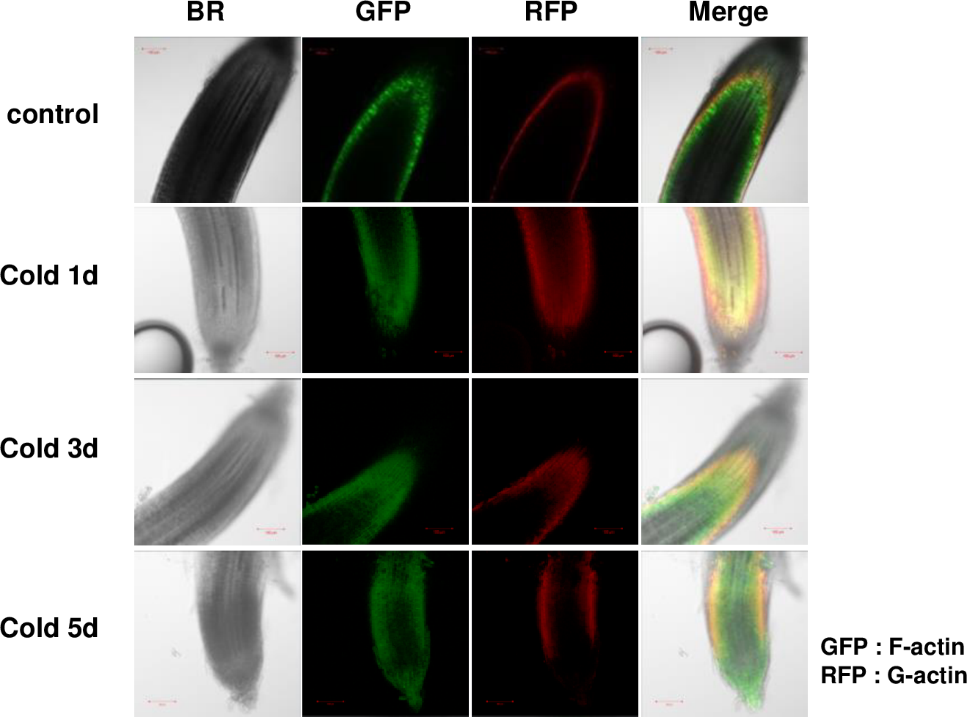


**Supplementary Figure 3.** Changes of actin dynamics in *D. antarctica* seedlings subjected to cold stress. Light-grown, 3-week-old *D. antarctica* were grown at 4°C for 5 days. Pieces of root tissue apices (1 cm) from cold stress-treated plants were sampled, fixed directly, and incubated in a mixture of Alexa Fluor 488-DNase I and Alexa Fluor 568-phalloidin. The stained samples were observed using a laser scanning confocal microscope. The G-actin and F-actin signals were excited by lasers at 488 (RFP) and 550 nm (GFP), respectively.

| **Supplementary Table 1**. The accession numbers of ADF homologs used in phylogenetic analysis | | | | | | |
| --- | --- | --- | --- | --- | --- | --- |
| **Clade** | **Family** | **Species** | **Symbol** | **Database** | **Identifier** | **Reference** |
| Bryophyte | Marchantiaceae | *Marchantia polymorpha* | Mapoly0001s0106.1.p | Phytozome | Mapoly0001s0106.1.p |  |
| Bryophyte | Marchantiaceae | *Marchantia polymorpha* | Mapoly0026s0075.1.p | Phytozome | Mapoly0026s0075.1.p |  |
| Bryophyte | Marchantiaceae | *Marchantia polymorpha* | Mapoly0149s0023.1.p | Phytozome | Mapoly0149s0023.1.p |  |
| Bryophyte | Funariaceae | *Physcomitrium patens* | Pp3c26_7930V3.1.p | Phytozome | Pp3c26_7930V3.1.p |  |
| Bryophyte | Sphagnaceae | *Sphagnum fallax* | Sphfalx01G071700.1.p | Phytozome | Sphfalx01G071700.1.p |  |
| Bryophyte | Sphagnaceae | *Sphagnum fallax* | Sphfalx05G052100.1.p | Phytozome | Sphfalx05G052100.1.p |  |
| Eudicotyledons | Brassicaceae | *Arabidopsis thaliana* | AtADF1 | Phytozome | AT3G46010.1 | Feng et al. 2006 |
| Eudicotyledons | Brassicaceae | *Arabidopsis thaliana* | AtADF2 | Phytozome | AT3G46000.1 | Feng et al. 2006 |
| Eudicotyledons | Brassicaceae | *Arabidopsis thaliana* | AtADF3 | Phytozome | AT5G59880.1 | Feng et al. 2006 |
| Eudicotyledons | Brassicaceae | *Arabidopsis thaliana* | AtADF4 | Phytozome | AT5G59890.1 | Feng et al. 2006 |
| Eudicotyledons | Brassicaceae | *Arabidopsis thaliana* | AtADF5 | Phytozome | AT2G16700.1 | Feng et al. 2006 |
| Eudicotyledons | Brassicaceae | *Arabidopsis thaliana* | AtADF6 | Phytozome | AT2G31200.1 | Feng et al. 2006 |
| Eudicotyledons | Brassicaceae | *Arabidopsis thaliana* | AtADF7 | Phytozome | AT4G25590.1 | Feng et al. 2006 |
| Eudicotyledons | Brassicaceae | *Arabidopsis thaliana* | AtADF8 | Phytozome | AT4G00680.1 | Feng et al. 2006 |
| Eudicotyledons | Brassicaceae | *Arabidopsis thaliana* | AtADF9 | Phytozome | AT4G34970.1 | Feng et al. 2006 |
| Eudicotyledons | Brassicaceae | *Arabidopsis thaliana* | AtADF10 | Phytozome | AT1G01750.1 | Feng et al. 2006 |
| Eudicotyledons | Brassicaceae | *Arabidopsis thaliana* | AtADF11 | Phytozome | AT3G45990.1 | Feng et al. 2006 |
| Eudicotyledons | Solanaceae | *Solanum lycopersicum* | SlADF1 | Phytozome | Solyc01g094400.3.1 | Khatun et al. 2016 |
| Eudicotyledons | Solanaceae | *Solanum lycopersicum* | SlADF2 | Phytozome | Solyc01g111380.3.1 | Khatun et al. 2016 |
| Eudicotyledons | Solanaceae | *Solanum lycopersicum* | SlADF5 | Phytozome | Solyc06g005360.3.1 | Khatun et al. 2016 |
| Eudicotyledons | Solanaceae | *Solanum lycopersicum* | SlADF7 | Phytozome | Solyc09g010440.3.1 | Khatun et al. 2016 |
| Eudicotyledons | Solanaceae | *Solanum lycopersicum* | SlADF8 | Phytozome | Solyc09g072590.3.1 | Khatun et al. 2016 |
| Eudicotyledons | Solanaceae | *Solanum lycopersicum* | SlADF9 | Phytozome | Solyc09g090110.3.1 | Khatun et al. 2016 |
| Eudicotyledons | Solanaceae | *Solanum lycopersicum* | SlADF10 | Phytozome | Solyc10g017550.3.1 | Khatun et al. 2016 |
| Eudicotyledons | Solanaceae | *Solanum lycopersicum* | SlADF11 | Phytozome | Solyc10g084660.2.1 | Khatun et al. 2016 |

| **Supplementary Table 1**. continued. | | | | | | |
| --- | --- | --- | --- | --- | --- | --- |
| **Clade** | **Family** | **Species** | **Symbol** | **Database** | **Identifier** | **Reference** |
| Liliopsida | Poaceae | *Deschampsia antarctica* | DaADF1 | NCBI | MW818093 | This study |
| Liliopsida | Poaceae | *Deschampsia antarctica* | DaADF2 | NCBI | MW818094 | This study |
| Liliopsida | Poaceae | *Deschampsia antarctica* | DaADF3 | NCBI | MW818095 | This study |
| Liliopsida | Poaceae | *Deschampsia antarctica* | DaADF4 | NCBI | MW818096 | This study |
| Liliopsida | Poaceae | *Deschampsia antarctica* | DaADF5 | NCBI | MW818097 | This study |
| Liliopsida | Poaceae | *Deschampsia antarctica* | DaADF7 | NCBI | MW818098 | This study |
| Liliopsida | Poaceae | *Deschampsia antarctica* | DaADF9 | NCBI | MW818099 | This study |
| Liliopsida | Poaceae | *Deschampsia antarctica* | DaADF10 | NCBI | MW818100 | This study |
| Liliopsida | Poaceae | *Oryza sativa* | OsADF1 | NCBI | XP_015622816.1 | Feng et al. 2006 |
| Liliopsida | Poaceae | *Oryza sativa* | OsADF2 | NCBI | XP_015630947.1 | Feng et al. 2006 |
| Liliopsida | Poaceae | *Oryza sativa* | OsADF3 | NCBI | XP_015632173.1 | Feng et al. 2006 |
| Liliopsida | Poaceae | *Oryza sativa* | OsADF4 | NCBI | XP_015632174.1 | Feng et al. 2006 |
| Liliopsida | Poaceae | *Oryza sativa* | OsADF5 | NCBI | XP_015631622.1 | Feng et al. 2006 |
| Liliopsida | Poaceae | *Oryza sativa* | OsADF6 | NCBI | XP_015637229.2 | Feng et al. 2006 |
| Liliopsida | Poaceae | *Oryza sativa* | OsADF8 | NCBI | XP_015644676.1 | Feng et al. 2006 |
| Liliopsida | Poaceae | *Oryza sativa* | OsADF9 | NCBI | XP_015645783.1 | Feng et al. 2006 |
| Liliopsida | Poaceae | *Oryza sativa* | OsADF10 | NCBI | XP_015613387.1 | Feng et al. 2006 |
| Liliopsida | Poaceae | *Oryza sativa* | OsADF11 | NCBI | XP_015618885.1 | Feng et al. 2006 |
| Liliopsida | Poaceae | *Triticum aestivum* | TaADF | Phytozome | Traes_5BL_B35A2E6B2.1 | Ouellet et al. 2001 |
| Liliopsida | Poaceae | *Triticum aestivum* | TaADF1 | Phytozome | Traes_6AL_6388DB675.1 |  |
| Liliopsida | Poaceae | *Triticum aestivum* | TaADF2 | Phytozome | Traes_5BL_1A6FFA35C.1 |  |
| Liliopsida | Poaceae | *Triticum aestivum* | TaADF3 | Phytozome | Traes_5BL_631FCC555.1 |  |
| Liliopsida | Poaceae | *Triticum aestivum* | TaADF4 | Phytozome | Traes_5DL_D9FB8D10D.1 |  |
| Liliopsida | Poaceae | *Triticum aestivum* | TaADF5 | Phytozome | Traes_4BL_5AF52F028.1 |  |
| Liliopsida | Poaceae | *Triticum aestivum* | TaADF7 | Phytozome | Traes_1BS_771984F00.1 |  |
| Liliopsida | Poaceae | *Triticum aestivum* | TaADF10 | Phytozome | Traes_1AL_31FEF29AE.1 |  |

| **Supplementary Table 1**. continued. | | | | | | |
| --- | --- | --- | --- | --- | --- | --- |
| **Clade** | **Family** | **Species** | **Symbol** | **Database** | **Identifier** | **Reference** |
| Liliopsida | Poaceae | *Zea mays* | ZmADF1 | Phytozome | Zm00001d021497 | Huang et al. 2020 |
| Liliopsida | Poaceae | *Zea mays* | ZmADF2 | Phytozome | Zm00001d006372 | Huang et al. 2020 |
| Liliopsida | Poaceae | *Zea mays* | ZmADF3 | Phytozome | Zm00001d034644 | Huang et al. 2020 |
| Liliopsida | Poaceae | *Zea mays* | ZmADF4 | Phytozome | Zm00001d035629 | Huang et al. 2020 |
| Liliopsida | Poaceae | *Zea mays* | ZmADF5 | Phytozome | Zm00001d028392 | Huang et al. 2020 |
| Liliopsida | Poaceae | *Zea mays* | ZmADF6 | Phytozome | Zm00001d013141 | Huang et al. 2020 |
| Liliopsida | Poaceae | *Zea mays* | ZmADF7 | Phytozome | Zm00001d051388 | Huang et al. 2020 |
| Liliopsida | Poaceae | *Zea mays* | ZmADF8 | Phytozome | Zm00001d047942 | Huang et al. 2020 |
| Liliopsida | Poaceae | *Zea mays* | ZmADF9 | Phytozome | Zm00001d034326 | Huang et al. 2020 |
| Liliopsida | Poaceae | *Zea mays* | ZmADF10 | Phytozome | Zm00001d012966 | Huang et al. 2020 |
| Liliopsida | Poaceae | *Zea mays* | ZmADF11 | Phytozome | Zm00001d029656 | Huang et al. 2020 |
| Liliopsida | Poaceae | *Zea mays* | ZmADF12 | Phytozome | Zm00001d002714 | Huang et al. 2020 |
| Liliopsida | Poaceae | *Zea mays* | ZmADF13 | Phytozome | Zm00001d017516 | Huang et al. 2020 |

| **Supplementary Table 2.** List of synthetic oligonucleotide sequences used in this study | | | | |
| --- | --- | --- | --- | --- |
| **Name** | **Sequence** | | **Purpose** | **Reference** |
|  | **Forward** | **Reverse** |  |  |
| DaEF1a | TTTGTCCACTGCTACACTCGTGGT | TCGAAGGCTGACGGACATAACCAA | RT-qPCR | Byun et al. 2015 |
| DaIRIP | AAGGGACCAACAACAATGTC | GACACCCAGTTACGATAGTG | RT-qPCR | Byun et al. 2015 |
| OsUbiquitin | ATGCAGATCTTTGTGAAGACATTG | TTACTGACCACCACGGAGGC | RT-PCR | Byun et al. 2015 |
| OsADF | ATGGCGAACGCGACGTCG | TTAGGAGGTGTGGTCCTTGAGCAC | RT-PCR | This study |
| DaADF1 | ATGGACATCGTTAAGGGGCG | TAGGCGAGTGTGGTTGTACG | RT-qPCR | This study |
| DaADF2 | TTCGTATGATGCCCAGAGCC | ACAGTAAGGGGCAGCTAGGA | RT-qPCR | This study |
| DaADF3 | GTGGTTGCTGTTCAGGTCCA | GCATCAGCGTCCCTTCTCTG | RT-qPCR | This study |
| DaADF4 | TGGTGGCGTGACTCTTGAAA | AGCCACGGAGACATTTCGAC | RT-qPCR | This study |
| DaADF5 | GTTTTATGTCCGCGTGCCTC | ATCGAAGCACAAACCCTCGT | RT-qPCR | This study |
| DaADF7 | GCTGGTCATGAGTTGTTGCC | TGCTGGCAGGTATGAGAGGA | RT-qPCR | This study |
| DaADF9 | CAGCGAGATGAGCCTTGACA | TGTGAAGCTGGAGTGTGTGT | RT-qPCR | This study |
| DaADF10 | CCCTGACGACATGGACTTGG | GTCTTGAGCTACCGGTGTGT | RT-qPCR | This study |

| **Supplementary Table 3.** Similarity analysis of ADF gene family of *D. antarctica* | | | | | |
| --- | --- | --- | --- | --- | --- |
| Gene Name | Top matched ADF clone | Name of matched protein | Identity (%) | E value | Matched species |
| DaADF1 | AGW22223.1 | actin-depolymerizing factor 4 | 96 | 3.0E-93 | *Triticum aestivum* |
| DaADF2 | XP_003558421.1 | actin-depolymerizing factor 2 | 95 | 2.0E-99 | *Brachypodium distachyon* |
| DaADF3 | AAC49404.1 | WCOR719 | 81 | 8.0E-82 | *Triticum aestivum* |
| DaADF4 | XP_010227656.1 | actin-depolymerizing factor 4 | 75 | 1.0E-72 | *Brachypodium distachyon* |
| DaADF5 | XP_020167342.1 | actin-depolymerizing factor 5 | 94 | 2.0E-96 | *Aegilops tauschii subsp. tauschii* |
| DaADF7 | XP_020173235.1 | actin-depolymerizing factor 7 | 98 | 1.0E-95 | *Aegilops tauschii subsp. tauschii* |
| DaADF9 | XP_037481475.1 | actin-depolymerizing factor 9 | 94 | 2.0E-91 | *Triticum dicoccoides* |
| DaADF10 | XP_003574212.1 | actin-depolymerizing factor 10 | 96 | 5.0E-99 | *Brachypodium distachyon* |

| **Supplementary Table 4.** Sequence identity among the 8 ADF proteins of *D. antarctica* | | | | | | | | |
| --- | --- | --- | --- | --- | --- | --- | --- | --- |
|  | DaADF1 | DaADF2 | DaADF3 | DaADF4 | DaADF5 | DaADF7 | DaADF9 | DaADF10 |
| DaADF1 | 100 |  |  |  |  |  |  |  |
| DaADF2 | 59 | 100 |  |  |  |  |  |  |
| DaADF3 | 55 | 44 | 100 |  |  |  |  |  |
| DaADF4 | 56 | 49 | 51 | 100 |  |  |  |  |
| DaADF5 | 55 | 54 | 46 | 45 | 100 |  |  |  |
| DaADF7 | 72 | 59 | 52 | 55 | 55 | 100 |  |  |
| DaADF9 | 71 | 52 | 49 | 53 | 48 | 63 | 100 |  |
| DaADF10 | 53 | 63 | 48 | 39 | 58 | 53 | 51 | 100 |
